# Bottom-up development of national obstetric guidelines in middle-income country Suriname

**DOI:** 10.1186/s12913-019-4377-6

**Published:** 2019-09-09

**Authors:** Kim J. C. Verschueren, Lachmi R. Kodan, Tom K. Brinkman, Raez R. Paidin, Sheran S. Henar, Humphrey H. H. Kanhai, Joyce L. Browne, Marcus J. Rijken, Kitty W. M. Bloemenkamp

**Affiliations:** 1 Department of Obstetrics, Division Women and Baby, Birth Centre Wilhelmina’s Children Hospital, University Medical Center Utrecht, Utrecht, the Netherlands; 2 Department of Obstetrics, Academical Hospital Paramaribo, Paramaribo, Suriname; 3 Julius Global Health, Julius Centre for Health Sciences and Primary Care, University Medical Centre Utrecht, Utrecht University, Utrecht, The Netherlands; 4 Department of Obstetrics, Diakonessen Hospital, Paramaribo, Suriname; 5 Anton de Kom University, Paramaribo, Suriname; 6 Department of Obstetrics, Department of Obstetrics, Leiden University Medical Center, Leiden, the Netherlands

**Keywords:** Clinical guidelines, Contextually-tailored guidelines, Locally adapted guidelines, Post partum hemorrhage, Hypertensive disorders of pregnancy, Middle-income country, Suriname

## Abstract

**Background:**

Obstetric guidelines are useful to improve the quality of care. Availability of international guidelines has rapidly increased, however the contextualization to enhance feasibility of implementation in health facilities in low and middle-income settings has only been described in literature in a few instances. This study describes the approach and lessons learned from the ‘bottom-up’ development process of context-tailored national obstetric guidelines in middle-income country Suriname.

**Methods:**

Local obstetric health care providers initiated the guideline development process in Suriname in August 2016 for two common obstetric conditions: hypertensive disorders of pregnancy (HDP) and post partum haemorrhage (PPH).

**Results:**

The process consisted of six steps: (1) determination of how and why women died, (2) interviews and observations of local clinical practice, (3) review of international guidelines, (4) development of a primary set of guidelines, (5) initiation of a national discussion on the guidelines content and (6) establishment of the final guidelines based on consensus. Maternal enquiry of HDP- and PPH-related maternal deaths revealed substandard care in 90 and 95% of cases, respectively. An assessment of the management through interviews and labour observations identified gaps in quality of the provided care and large discrepancies in the management of HDP and PPH between the hospitals. International recommendations were considered unfeasible and were inconsistent when compared to each other. Local health care providers and stakeholders convened to create national context-tailored guidelines based on adapted international recommendations. The guidelines were developed within four months and locally implemented.

**Conclusion:**

Development of national context-tailored guidelines is achievable in a middle-income country when using a ‘bottom-up’ approach that involves all obstetric health care providers and stakeholders in the earliest phase. We hope the descriptive process of guideline development is helpful for other countries in need of nationwide guidelines.

**Electronic supplementary material:**

The online version of this article (10.1186/s12913-019-4377-6) contains supplementary material, which is available to authorized users.

## Plain English summary

One of the most important steps to reduce maternal deaths in low and middle-income countries (LMIC) is improving the quality of care provided to pregnant women. Guidelines are known to improve the quality of care, but international guidelines are often not directly applicable for LMIC due to the lack of personnel or resources. This in part reflects where and by whom the guidelines are often developed: international organizations, international colleagues or non-clinicians, who insufficiently consider the context in which these guidelines need to work.

In Suriname, a South American middle-income country, local health care providers initiated a guideline development strategy themselves (“bottom-up”). Local health care providers developed two obstetric guidelines about severe bleeding after childbirth (postpartum haemorrhage) and high blood pressure (hypertensive disorders) during pregnancy. This process consisted of a number of steps: first, it was studied how and why women died during pregnancy, childbirth or shortly after. Next, interviews with local health care providers were conducted, local clinical practice was observed, international guidelines were reviewed and the preliminary guidelines were developed. These guidelines were discussed nationally and consensus was reached for the final guidelines after which implementation began. This “bottom-up” approach, with early involvement of local health care providers proved essential to develop guidelines that are acceptable to everyone and guarantee implementation. These lessons can inform and support the guideline development processes in other countries.

## Background

Reducing maternal mortality remains a universal priority for clinicians, researchers and policymakers. The obstetric transition model describes five stages in which countries move, from high to low maternal mortality [1]. Phase three is considered a tipping point, in which predominantly direct causes of mortality persist, but as most women reach hospitals, improving the quality of care (skilled birth attendance, appropriate management of complications) becomes essential to further reduce mortality [1]. In low- and middle-income countries (LMIC), where informal sharing of knowledge and experience-based decision-making often dominates, the development and implementation of feasible clinical guidelines are key to improve quality of evidence-based, respectful maternity care [2].

Evidence about guideline implementation strategies in low- and middle-income countries has increased in the past years and a number of enablers of effective impementation have been identified [3, 4]. The most important known enabler is to use a multi-facetted strategy (i.e. combining different methods of implementation) instead of a single intervention (e.g. providing health care workers with existing guidelines) [3–7]. Positive health care providers’ attitude towards the guidelines is strongly associated with adherence to the guidelines. The process of guideline development *before* implementation is critical. By creating appropriate guidelines tailored to the context, use in local reality is ensured and sustainable adherence is created [7–9].

Suriname is an example of a country in obstetric transition phase three with a fairly high maternal mortality ratio (MMR) of 130 per 100.000 live births compared to other countries in Central and South America [10–12]. Similar to most LMICs, the primary causes of maternal deaths in Suriname are postpartum hemorrhage (PPH) and hypertensive disorders of pregnancy (HDP) [11]. The majority of these deaths are due to ‘third delay’ factors linked to the quality of care, such as in-hospital delay of diagnosis and treatment [11, 13]. The introduction of nationwide guidelines for the clinical management of these complications is therefore a promising strategy to improve health outcomes.

The aim of this article is to describe the approach and lessons learnt from our *‘bottom-up’* strategy to develop national guidelines tailored to the context of middle-income country Suriname for post partum hemorrhage and hypertensive disorders of pregnancy. These lessons can inform and support the guideline development processes in other settings.

## Methods

Suriname is a middle-income country on the northeastern coast of South-America with 550.000 inhabitants in 2016 and almost 10.000 deliveries annually. Of all deliveries, 92% of women give childbirth in the five hospitals in the country, while 8% deliver in primary health care centers and 2% at home. Four hospitals are located in capital city Paramaribo and one smaller hospital is located on the far West-coast, Nickerie [14, 15]. In 2016, fifteen obstetricians, eight residents and approximately fifty midwives provided maternal care in the hospitals in Suriname. Obstetric care provision in Suriname is mainly influenced by Dutch guidelines (Nederlandse Vereniging van Obstetrie en Gynaecologie, NVOG) as residents follow 2 years of their training in the Netherlands [16, 17]. In addition, the American College of Obstetrics and Gynecology (ACOG) and the World Health Organization (WHO) guidelines are used [18–21].

In 2015 and 2016 a national maternal death review committee was established, consisting of local obstetric health care providers. This committee audits all pregnancy-related deaths in the country. Among the recommendations are the implementation of national guidelines on the most important causes of maternal mortality and training emergency (obstetric) skills. Subsequently, the maternal death committee members initiated the bottom-up guideline development consisting of six-steps, as described below.

### Determine how and why women died

A Reproductive Age Mortality Survey was initiated by a local obstetrician (L.K.) and the principle investigator (K.V.) to audit all maternal deaths between 2010 to 2014. The study revealed a maternal mortality ratio of 130 per 100.000 live births with many preventable deaths due to post partum hemorrhage and hypertensive disorders [11]. The maternal deaths due to, or aggravated by HDP and PPH were further analyzed for substandard care factors and the three-delay model was applied to establish why women died and what could have prevented the death [22].

### Interviews and observations of local clinical practice

First, to determine the standard of care for HDP and PPH management, the obstetric departments of the five hospitals were asked to share their local protocols. Second, interviews on practice were performed with forty-three obstetric health care providers from all hospitals: 13 obstetricians, 8 residents, and 24 midwives. An anonymous national questionnaire was completed. The questionnaire was developed for the purpose of this study (see Additional file 1). The structure of the interview was based on international consensus on HDP and PPH prevention, diagnosis and treatment (adapted ACOG checklists) [19, 23]. Questions were also asked on encountered barriers and enablers in the current system. Semi-structured one-on-one interviews, conducted by the principle investigator (KV), were held with the gynaecologists and head of midwives of each hospital to assess their opinions and wishes with regard to the new guidelines.

Third, clinical observations were performed by four medical doctors (LH, TB, RP, SH) working in the hospitals and four medical students conducting their rotations. The principle investigator provided the observers a summary of the abovementioned findings per hospital. During a two-month period (250 deliveries) observations were performed in all hospitals on whether the answers in the surveys matched reality. The medical students used the ACOG-adapted checklists for HDP and PPH during the observations.

### Review international guidelines

The four international guidelines on HDP and PPH used most by local health care providers were compared for similarities and differences in definition, causes and recommendations in diagnosis and treatment. Both the HDP and PPH guidelines from the WHO, ACOG and NVOG were assessed. Additionally the PPH guideline of the British Royal College of Obstetrics and Gynaecology (RCOG) and HDP guideline of Australian Queensland Brisbane (QB) were assessed.

### Develop a primary set of guidelines

In August and September 2016 the initial version of the guidelines were drafted by four members of the study team (KV, LK, TB, SH) and one nurse-midwife of each hospital. The above mentioned guidelines were used as a template.

The drafted guidelines were reviewed by external (four international experts from the Netherlands, of which authors HK and KB) and internal reviewers (eighteen local obstetricians and nurse-midwives). The reviewers independently discussed the guidelines with the principle investigator. During a three-hour meeting with all the reviewers the ‘key discussion points’ were established and simulation-based trainings were prepared. A literature search was conducted on the ‘key discussion points’ by five of the authors (KV, TB, RP, LK, KB) for evidence-based answers and considerations.

### Initiate national discussion about content of guidelines

Two hundred and one obstetric health care providers (obstetricians, paediatricians, anesthesiologists, residents, doctors, midwives, nurses, trainees) and policy makers (Ministry of Health and Pan-American Health Organization (PAHO)) attended a four-day conference (November 10th to 13th 2016) to discuss the recommendations in two new national obstetric guidelines. The meeting was moderated by one local and one international obstetrician. The two guidelines were adapted during the conference. A two-hour simulation-based training was held on each day to practise and evaluate the content of the guidelines. These trainings were based on maternal deaths of the previous years and led by a team existing of one international expert, two local obstetricians, an anesthesiologist and two midwives. The participants completed an evaluation survey (5-point Likert scale, from unsatisfied to extremely satisfied) about the different components of the conference.

### Final guideline development and evaluation

The last drafts of the guidelines were distributed digitally and on paper. All obstetric health care providers (including those who did not attend the meeting) were requested to provide feedback within 6 weeks. The local obstetricians (*n* = 13) and chief midwives of each hospital (*n* = 5) were personally visited for the final feedback and their formal approval.

Ethical approval was obtained from the Surinamese Central Committee on Researching Human Subjects for the study of maternal deaths [Reference number VG 006–15] in March 2015 [11]. The Surinamese Central Committee on Researching Human Subjects waived the need for approval for the remainder of the project.

## Results

**Fig. 1:**
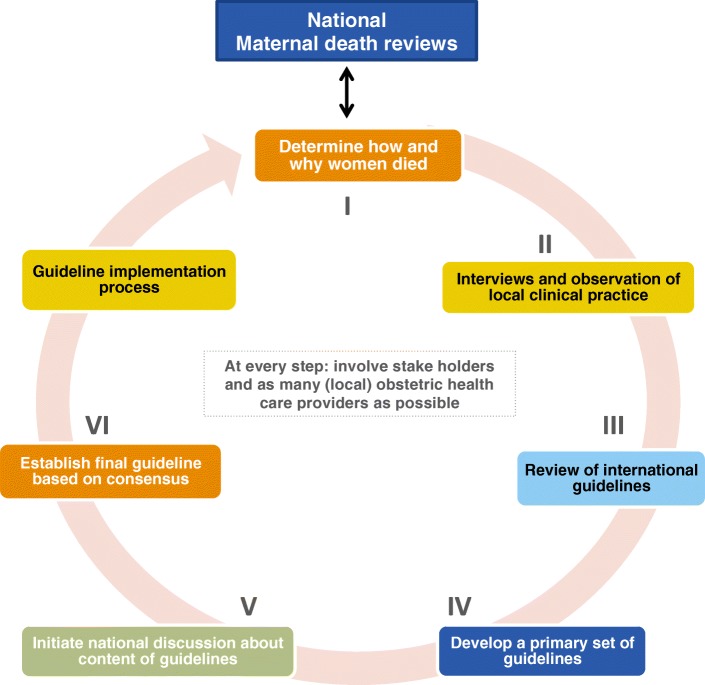
Obstetric guideline development strategy in Suriname in six steps

The six phases of the guideline development process were executed in a period of four months (Fig. 1).

### Determine how and why women died

The substandard care analysis of the extracted maternal deaths related to HDP (*n* = 19) and PPH (*n* = 21) in Suriname reveals that most substandard care factors are *third delay* factors due t a lack of quality of care (see Table 1) [11].

**Table 1:** Three-delay model of maternal deaths in Suriname, 2010–2014

	HDP-related maternal deaths *n* = 19 (%)	PPH-related maternal deaths *n* = 21 (%)
1st delay (patients do not seek care)	1 (5.3)	1 (4.8)
2nd delay (patients do not reach care)	1 (5.3)	4 (19.0)
3rd delay (patients do not receive adequate care in hospital), reasons:	17 (89.5)	20 (95.2)
i. Essential medications unavailable	0 (−)	2 (10.0)
ii. Blood products unavailable	N/A	3 (15.0)
iii. Necessary staff unavailable	1 (5.9)	2 (10.0)
iv. Lack of quality of care (delay in diagnosis and treatment (not due to unavailability), inadequate monitoring, poor supportive treatment.	16 (94.1)	19 (95.0)
Death most likely preventable	9 (47.4)	16 (76.2)

The substandard quality in care seen in the maternal deaths was mostly due to the lack of anticipation, delay in recognition of seriously ill women, delay in providing available treatment and a lack of supportive treatment (e.g. oxygen, uterine massage). In three (15%) of the maternal deaths blood products were not available within 60 min. Medication (oxytocin, misoprostol or magnesium sulfate) was not available in two women who died of PPH during transportation to the hospital.

### Interviews and observations of local clinical practice

Two of the five hospitals used short local protocols for HDP and PPH. These protocols were available both digitally and on paper. The other three hospitals did not have protocols.

Fourty-three interviews were conducted with obstetric health care providers and showed differences in perception of optimal HDP (Table 2) and PPH-care (Table 3). The interviewees indicated that the guidelines most frequently used by local staff were from WHO, NVOG and ACOG, though only 60% (*n* = 26/43) said to actually use them [16–21]. A frequent mentioned argument of the health care providers was that the international guidelines were *“complex, not applicable, not achievable, unclear and/or not practical in its use”*. Doctors mentioned that *“there are discrepancies between international guidelines leading to discrepancies in regimens between gynaecologists in daily practice”*. Lastly, midwives mentioned that they *“miss easy-to-use checklists or flowcharts which we can adapt to our situation”*.

**Table 2:** Interviews with obstetric health care providers on the standard local care regarding HDP

Hypertensive disorders of pregnancy	*n* = 43 (100%)
Definitions clear	
- Pre-eclampsia	38 (88)
- Severe pre-eclampsia	22 (51)
- Eclampsia	34 (79)
Anticipation / prevention	
- Risk factors known	36 (84)
- Aspirine	10 (23)
- Calcium	22 (51)
Oral medical treatment (1^st^ line)	
1. Methyldopa	43 (100)
2. Hydralazine	43 (100)
3. Nifidipine (antepartum)	5 (12)
4. Labetalol	21 (48)
Parenteral medical treatment (2^nd^ line)	
1. Hydralazine (direct shots)	43 (100)
2. Hydralazine (perfussor)	25 (58)
3. Labetalol	15 (35)
4. Ketanserin	5 (12)
Magnesium sulfate	
- Loading dose (4–6 g/30 min)	26 (60)
- Maintanance dose (1 g/hr)	43 (100)
- Initiation threshold: BP ≥110	32 (74)
- Duration: 24 – 48 hours	43 (100)
- Repeat (2 g/5 min) in seizure	6 (14)
- Diazepam before MgSO_4_	38 (88)
Stabilization of severe PE / eclampsia	
- Minimum 48 hr before termination of pregnancy	8 (19)
Earliest termination in severe PE	
- GA ≥ 27 weeks	23 (53)
- GA ≥ 30 weeks	15 (35)
- GA ≥ 32 weeks	5 (12)
Other	
- Eclampsia box available^a^	9 (21)
- Oxygen during eclampsia	12 (28)
- Two i.v. access lines	22 (51)
- i.v. loading fluid before MgSO4	7 (16)
- Restrict fluids to < 2 L / 24 hrs	0 (0)
- Early warning score (MEOWS)	7 (16)

^a^Eclamspia kit includes magnesium sulfate, calciumgluconate, labetalol, hydralazin, sodium choloride ampoule, fluids (ringers lactate and sodium chloride), blood sample bottles, tourniquet, syringes, plaster to fix cannula, guedel aiways, bag and mask, oxygen, reflex hammer

**Table 3:** Interviews with obstetric health care providers on the standard local care regarding PPH

Post partum hemorrhage	*n* = 43 (100%)
Definitions clear	
PPH	38 (88)
Severe PPH	19 (44)
Clear when to alarm doctor	24 (56)
Anticipation / prevention	
Uterotonics in Caesarean	43 (100)
Uterotonics in all vaginal births	14 (33)
Controlled cord traction	19 (44)
Measuring blood	
Measuring by cup	18 (42)
Only clots measured	16 (37)
Medical treatment (1^st^ line)	
Oxytocin i.m. or i.v. (2nd shot)	16 (37)
Oxytocin infusion (10IU/4 hrs)	43 (100)
Misoprostol 400mcg supp	41 (95)
Methergine 0.2mg i.m.	11 (26)
Resuscitation	
Always place 2nd i.v. line	11 (26)
Choice of fluids:	
Crystalloids	43 (100)
Colloids	16 (37)
Oxygen	20 (47)
Tranexamic acid (1 gr i.v.)	10 (23)
Blood transfusion	
Clear guidelines available	0 (0)
Indication:	
Hb < 4 mmol/L	37 (86)
Hb < 3.5 + Ht <0.20	6 (14)
Persistent blood loss	43 (100)
Ratio:	
1 PC : 2 FFP	9 (21)
2 PC : 1 FFP	34 (79)
Other	
PPH box available^a^	19 (44)
Balloon / B-Lynch / uterine pack	0 (0)
Vaginal tampon	43 (100)
Hysterectomy if necessary	43 (100)
Early warning score (MEOWS)	9 (21)

^a^PPH box includes oxytocin, Methergin, misoprostol, different IV cannulas, blood sample bottles, tourniquet, syringes, plaster to fix cannula, catheter size 16 with urobag, infusion set, blood set, sterile gloves, cotton swabs, scissors, fluids (ringers lactate and sodiumchloride), 3-way connectors, oxygen face mask, spculums, sponge holding forceps, condom tamponade and catheter, uterine pack

The ‘standard care’ regarding HDP and PPH per hospital can be found in Additional file 2. Noteworthy, are differences in daily regimen between hospitals within five kilometers distance of each other. Clinical observations revealed that the management of HDP and PPH was found to differ between obstetricians within the same hospital (in three hospitals).

**Fig. 2:**
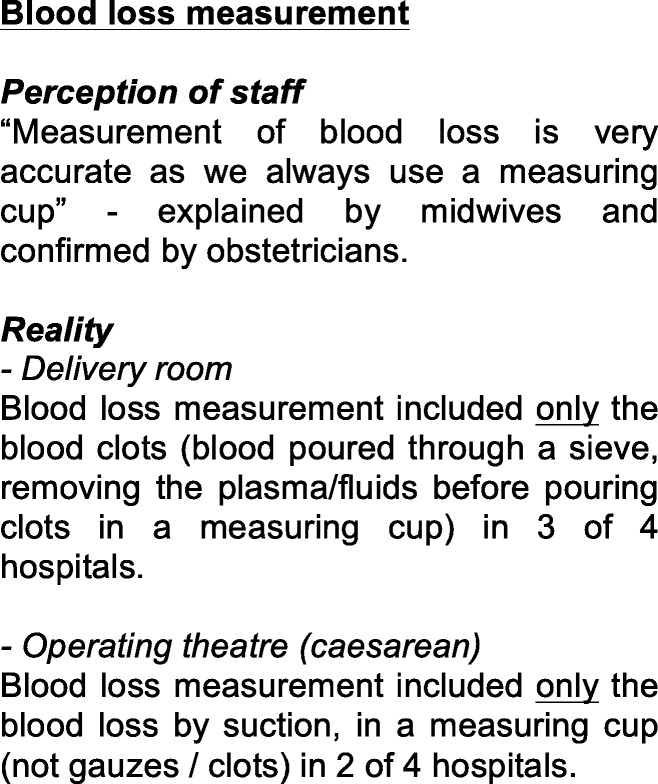
Example of revelations during clinical labor observations

We did not observe that the international guidelines were consulted by nurses or midwives in any of the hospitals. When asked for, the digitally available protocols could not be localized by the staff on duty in two of the hospitals. In PPH management, we observed blood loss estimation was often inadequately (Fig. 2).

### Review international guidelines

A summary of similarities and differences between the four international guidelines used most frequently by local health care providers is presented in Additional file 3.

The four HDP guidelines (WHO, ACOG, QB, NVOG) differed from each other in the following major recommendations: diagnosis of severe pre-eclampsia, timing of aspirin prevention, antihypertensive therapy choices and dose, magnesium sulfate dose and therapy duration, recommendations on fluid restriction, vital sign monitoring and timing of delivery [17, 18, 21, 24].

The four PPH guidelines (WHO, ACOG, RCOG, NVOG) differed from each other in the following major recommendations: active management in the third stage of labour (AMTSL), dose of uterotonics, oxygen therapy initiation, uterine massage, blood transfusion ratio, tranexamic acid, balloon tamponade, embolization, vessel ligation and hysterectomy [16, 19, 20, 25].

### Development of the two national guidelines

The first drafts of the guidelines were created in September 2016. International reviewers added specific recommendations, e.g. the use of tranexamic acid in PPH, the necessity of a blood transfusion protocol, restricting fluids in pre-eclampsia and aspirin prevention of pre-eclampsia. The local reviewers (all gynecologists and head nurses) requested for the guidelines to be more practical with flowcharts and checklists and with more specific recommendations, e.g. the frequency of vital sign monitoring in HDP or PPH. The ‘key discussion points’ were summarized and attached to the draft guidelines as an appendix.

### Initiate national discussion about content of guidelines

The four-day meeting was attended by 201 health care providers, including all obstetricians (*n* = 15), residents (*n* = 4), the majority of midwives and nurses (−in training) (80%, *n* = 161)) and different stakeholders, i.e. representatives of the Ministry of Health and the Pan-American Health Organization. Key discussion points were presented and discussed for final consensus on the HDP (Table 4) and PPH guideline (Table 5). This discussions was facilitated by the local and international moderators who were prepared with evidence-based background information.

**Table 4:** ‘Key discussion points’ and the consensus reached during the HDP guideline development process

Hypertensive disorders of pregnancy “Key discussion points”		Consensus
Definition		
1	Can you diagnose severe pre-eclampsia without proteinuria?	*Yes, if severe hypertension is accompanied by hematologic, renal, neurological, hepatic or pulmonary complications* [26, 27].
Prevention		
2	Which women should receive aspirin therapy for the prevention of pre-eclampsia?	*All women with an obstetric history of pre-eclampsia should be administered 100mg of aspirin during their 16th and 37th week. Women with cardiovascular risk factors may be counselled for its use as well* [26, 28].
Therapy		
3	Which antihypertensive therapy is preffered?	*Methyldopa is first choice, followed by hydralazine or labetalol. Nifedipine is first choice postpartum.*
4	In severe HDP, what should be given first: antihypertensives or magnesiumsulfate?	*Magnesium sulfate (4 gram in 30 minutes, followed by 1 gram per hour) is initiated immediately; hypertensive medication will be added depending on the blood pressures.*
5	Can magnesium sulfate be administered by nurses or midwives according to protocol in eclampsia prior to consultation with a doctor?	*In emergency situations nurses or midwives can administer magnesium sulfate before or during consultation with a doctor. It should never be administered over the same tap as oxytocin.*
6	Is magnesium sulfate therapy without a loading dose an option when severe pre-eclampsia presents without clinical symptoms?	*International evidence and recommendations suggest always using a loading dose, as the maintenance dose does not give the magnesium plasma rise that is necessary to prevent a seizure* [29]. *When magnesium sulfate is given, it should be given adequately and not stopped during (caesarean) delivery.*
7	Should magnesium sulfate therapy be continued in caesarean section with spinal analgesia?	*Magnesium sulfate should be continued during the delivery or caesarean section, as the intra partum risk of eclampsia is highest* [26, 28].
8	Can nifedipine and magnesium sulfate therapy be combined?	*There is a potential theoretical interaction between the two, leading to hypotension and neuromuscular blockade effects, although this is seldom reported. Regular monitoring is recommended and if hypotension occurs, nifedipin and magnesium sulphate administration should cease* [30].
9	Should a fluid preload be administration before intravenous antihypertensive or magnesium sulfate therapy?	*No, because the risk of fluid overload (and subsequent pulmonary oedema) is high in severe pre-eclampsia* [26]. *Fluid preloading is acceptable in hypovolemia or anticipated epidural anaesthesia and low blood pressures. Early consultation of an anaesthesiologist is advised.*
10	Is diazepam of added value to magnesium sulfate in the treatment of eclampsia?	*Magnesium sulfate is the drug of choice for treating eclamptic seizures. Diazepam is not advised by international guidelines* [26]. *During the discussion it was decided upon that diazepam use should be limited to unremitting seizures.*
Other		
12	How can we define “stabilization” in eclampsia or severe pre-eclampsia?	*The definition of stabilization of pre-eclampsia and eclampsia is: (1) stable blood pressure (RR 130-150 / 70-100); (2) adequate magnesium sulfate (loading dose must be administered); (3) platelets of >80); (4) fluid restriction of <1.5 liters. Stabilization is not time-dependent and can be reached even within an hour if management is adequate* [26].
13	When and how should the pregnancy be terminated in eclampsia or severe pre-eclampsia?	*The delivery should not be terminated until the mother is stable (see point 13). The termination of pregnancy is on maternal indication. Vaginal delivery should be strived for whenever feasible without fetal compromise* [26].
13	How often should vital signs be checked and what should be checked?	*Every 15 minutes (4 times), every 30 minutes (4 times), every hour (4 times), every two hours (4 times), followed by regular checks in case of normal blood pressures.*
14	When is admission to the Intensive Care Unit indicated?	*In any case of organ dysfunction, such as pulmonary oedema, neurological complications, HELLP, etc.*

**Table 5:** ‘Key discussion points’ and the consensus reached during the PPH guideline development process

Post partum hemorrhage “Key discussion points”		Consensus
Definition		
1	Should the threshold for PPH be blood loss of 500mL (WHO) or 1000mL (Netherlands)?	*Blood loss after vaginal delivery is often underestimated. Therefore, blood loss of 500mL will be considered PPH.*
2	How should the blood loss be measured, by a measuring cup, by weight or estimation)	*Blood loss needs to be measured by measuring cup or by weight (minus the pad). To be precise, it is advised to exchange the pads just after childbirth to subtract the amniotic fluid loss.*
Prevention		
3	Should oxytocin prevention after childbirth always be available and given, including in rural areas?	*The cost-efficacy was discussed and health care workers of the interior (n=12) and the different stake holders agreed that oxytocin should be made available in the interior. Misoprostol use in the interior is avoided as much as possible to avoid unsafe abortion.*
4	Can the oxytocin-infusion used for uterine stimulation be used as preventive measure for PPH or is an extra bolus of oxytocin needed?	*A calculation of the blood oxytocin concentration after bolus or infusion revealed that an extra (intravenous or intramuscular) 5-10 units of oxytocin bolus is necessary for adequate prevention op PPH based on international recommendation* [31, 32, 33].
5	Which health care providers should be permitted to perform controlled cord traction?	*Midwives should all be competent in performing controlled cord traction. If they do not feel competent to do so, they should be trained by more experienced personnel.*
Therapy		
6	Misoprostol is frequently used in PPH in Suriname, what is the additional value on top of adequate oxytocin infusion?	*If oxytocin is adequately administered (an extra shot of 5 units plus continuous infusion of 10 units in maximum four hours), misoprostol has no additional value* [32]. *If oxytocin is not available, or the uterus does not contract sufficiently, misoprostol can be given.*
7	What should the oxytocin regimen be in caesarean section?	*Oxytocin 5 units slowly intravenously, followed by an infusion (10 units in four hours) is advised in all caesarean sections* [33].
Fluids and blood products		
8	In severe PPH should crystalloids or colloids be used?	*International recommendations show no better outcome when using colloids* [34]. *Colloids are more expensive, adverse effects have been reported and there is no decrease in the risk of respiratory problems due to pulmonary oedema* [35]. *The preference is to use crystalloids.*
9	What is the ideal ratio for the transfusion of packed cells, fresh frozen plasma and platelets?	*Ratio 1 : 1 : 0. For every packed cell also fresh frozen plasma. In acute severe blood loss it is advised to initiate the fresh frozen plasma transfusion, as it is generally available more rapidly than packed cells. Platelet transfusion is given on indication (coagulopathy).*
Other		
10	Should a parthograph always be used?	*The partograph is an important tool to assess the progress of labour. Induction or slow progress of labour and oxytocin-stimulation are merely a few examples of PPH risk factors.*
11	When is tranexamic acid recommended and what is the risk for a subsequent thrombo-embolism?	*Tranexamic acid (1 gram in 10 minutes) is recommended in cases of > 1000 mL blood loss. In on going blood loss, it is advised to repeat this after 30 minutes* [36]. *It should not be administered to women with a contra-indication for antifibrinolytic therapy (e.g. thrombosis in pregnancy). The results of the WOMAN trial are to be published.*
12	What are more affordable options for an intra-uterine tamponade balloon such as the Rush or Bakri?	*Intra-uterine balloon can be made with condoms and a urinary catheter. Recommendations were to insert a large vaginal tampon after the balloon insertion to prevent displacement.*
13	How often should vital signs be monitored after severe PPH?	*Every 5 to 10 minutes during blood loss. After PPH vitals should be recorded after 30 minutes, one hour, 2 hours, 4 hours and 8 hours.*

The simulation trainings in smaller groups were well-received as the local health care providers felt they *“could practice”* and felt *“safe to ask remaining questions”.* The evaluation survey revealed that the majority (93%, *n* = 186/201) of the health care providers were ‘very satisfied’ (4 or 5 points on scale of Likert) with the guideline development proces. There was a high rate of agreement on the content of the guidelines and commitment to implementation (82%, *n* = 164/201). The participants mentioned that they felt important to the development process*.* One fifth of the participants (18%, *n* = 37) commented that they would have liked more training opportunities and a better location for the four-day meeting.

### Final guideline development and evaluation

The obstetric health care providers had no further comments 6 weeks after guideline distribution. The final versions of both guidelines were approved by all obstetricians, head midwives and participating stakeholders. The final guidelines were distributed 4 months after the initiation of the project and further implementation followed in the hospitals. The Ministry of Health technically supported the abovementioned development process and accepted the guidelines as national guidelines. The guidelines were reviewed by health providers 2 years after initial implementation during a second national conference, and were adapted with new recommendations accordingly.

## Discussion

We have presented the participatory approach of the development process of context-tailored national obstetric guidelines on HDP and PPH in Suriname. The process consisted of six steps: (1) determination of how and why women died, (2) interviews and observations of local clincal practice, (3) review of international guidelines, (4) development of a primary set of guidelines, (5) initiation of a national discussion about the guideline content and (6) consensus-based finalization of both guidelines.

The most important enabler of succesful guideline development was the bottom-up approach with early involvement of local, intrinsically motivated, health care providers. Important barriers were the inconsistencies between international recommendations, the unavailability of easily adaptable guidelines and the use of several different international guidelines by health care providers which differed among each other.

In the assessment of causes of maternal deaths due to HDP and PPH in Suriname, we found that insufficient quality of care played the most important role. This led to the development of the guidelines. Our approach is aligned with the recommendations from the obstetric transition model, in which evidence-based guideline implementation is a key intervention to further reduce maternal mortality in countries in the third stage of transition [1, 37, 38]. Contrary to the more common maternal health guideline development approach (with a top-down mentality, in which experts distribute knowledge or guidelines without involvement of target users), our guideline development approach demonstrates how to succesfully bridge the gap between evidence-based international recommendations and local realities by involving end users from the earliest phases of guideline development to enhance final guideline use [3, 4, 8, 38]. This is crucial, as merely the existence of (international) guidelines does not guarantee implementation. Our assessment of practice in the hospitals in Suriname showed that guidelines for HDP and PPH were not routinely used and quality of decision-making was based on experience rather than evidence, as often reported from similar settings [5, 7, 8, 39]. Local health professions often considered the international guidelines unfeasible and impractical. Next to this, similar to other studies, we found that well-established international guidelines on HDP and PPH differ significantly in their recommendations and interpretation of underlying evidence that resulted in these recommendations [40–42]. This suggests that a critical evaluation is necessary of how available evidence is used to develop global obstetric guidelines.

There were substantial differences in the management of HDP and PPH between hospitals. This is in part a reflection of the various clinical practices that influence care in Suriname with influences from Europe, the United States and the WHO. Yet, even in high-income countries with national guidelines endorsed by professional organizations, inter-hospital differences in management of HDP and PPH are reported [43, 44]. These findings underline the importance of involving also the end users in the guideline development process (i.e. health care providers and stakeholders involved in pregnancy, delivery and postpartum care).

Important barriers of guideline development that need to be elaborated upon is that some recommendations are not immediately accepted. In Suriname, for example, the use of magnesium sulfate for prevention of eclampsia was initially not accepted by all as healthcare workers were not yet familiar with this. Another barrier for Suriname is the fact that there is currently no regulatory framework for health professionals. We believe such framework would be relevant to reduce maternal deaths, especially by helping health care workers to monitor their delivered quality of care. A general barrier for the development of context-tailored obstetric guidelines is the fact that it is time consuming and resource demanding. We noticed that early involvement of end users, understanding their barriers and engaging all health care professionals are essential to ensure a fast guideline development process.

If more global consensus on the most important obstetric complications would be attained, with recommendations tailored at region or health care system resources and include easily-adaptable flowcharts or checklists, the local guideline development would be much more feasible. The WHO Handbook for Guideline Development is an example of a comprehensive tool for evidence-based guideline development, but also a very large document and it uses a time-consuming process that seems not readily achievable for most LMIC [45]. The recently published PartoMa study from in a low-resource referral hospital in Zanzibar, Tanzania, is one of the few examples of a systematic approach to evidence-based international recommendations adaptation to local reality and evaluation of it’s impact on health outcomes. Their ‘bottom up’ approach was similar to ours and appeared to be associated with significant reductions in stillbirths and improvement of treatment of hypertensive disorders of pregnancy [6, 7, 46]. The PartoMa Guideline Development in Zanzibar and our strategy in Suriname were both achievable due to the smaller size of the island or country [6, 7, 46]. However, when healthcare workers are fully engaged in the quality cycle of plan-do-check-act, nationwide improvement can also be made in larger countries [47].

### Strengths and limitations

This is the first evaluation of the development of national obstetric guidelines in a middle-income country, and can serve as an example for low and middle-income countries in the process of developing contextually-tailored guidelines. There was a high rate of agreement and the guideline development process was completed very steadily, in only 4 months.

A limitation to consider is that our guideline development process was conducted in a small country and thus, for larger countries this might not be applicable directly. Another limitation is that qualitative data on maternal mortality most likely did not reflect ‘standard’ management of HDP and PPH before the guideline development proces. We therefore recommend others to perform a study before guideline development to better assess the current situation and be able to do a before-after analysis. We also acknowledge that the evaluation surveys were not conducted by an independent party and may not have captured all the dimensions of the development process. Nevertheless, if the incidence of maternal mortality and severe morbidity declines, the awareness created among healthcare providers by recent publications on local maternal mortality in Suriname together with the development of the guidelines will likely have contibuted, especially as no other major interventions related to HDP and PPH have taken place the past decade [11, 13]. In the context of research, we are evaluating implementation of the guidelines by criteria-based audits embedded in a prospective cohort study on severe maternal morbidity and mortality due to HDP and PPH, currently ongoing in Suriname. Yet, it remains a recommendation to independently evaluate the impact on core outcomes in order to evaluate actual quality improvement.

## Conclusion

Bottom-up development of context-tailored guidelines are achievable within a reasonably short timeframe. Important barriers for the guideline development process are the discrepancies between international recommendations, which require local consensus to be reached on key issues, and the unavailability of easily adaptable guidelines. The main enabler for both development and implementation is the involvement of local birth attendents from the early phases onwards to ensure use in local reality, drive change and create sustainable adherence. We recommend bottom-up context-tailored guideline development with early involvement of the end users.

## Additional files

Additional file 1: Questionnaire on HDP and PPH practice in your facility. (PDF 108 kb)

Additional file 2: Comparison of hospitals in clinical practice of HDP and PPH in Suriname. (PDF 124 kb)

Additional file 3: Comparison of international guidelines regarding HDP and PPH. (PDF 124 kb)

## Data Availability

The datasets analysed during the current study are not publicly available due to the sensitivity of the data, but are available from the corresponding author on reasonable request. The final guidelines can be found on verloskundesuriname.org

## Abbreviations

ACOG: American College of Obstetrics and Gynaecology
CCT: Controlled cord traction
FFP: Fresh frozen plasma
Hb: Hemoglobin
HDP: Hypertensive disorders of pregnancy
HIC: High income country
IU: International Units
LMIC: Low and middle income country
MEOWS: Modified Early Obstetric Warning Score
NVOG: Nederlandse Vereniging van Obstetrie en Gynaecologie
PAHO: Pan-American Health Organization
PC: Packed cell
PPH: Post partum hemorrhage
QB: Queensland Brisbane
RCOG: Royal College of Obstetrics and Gynecology
WHO: World Health Organization
