# Supplementary material for: Bottom-up development of national obstetric guidelines in middle-income country Suriname
Source: BMC Health Serv Res. 2019 Sep 9;19:651. doi: 10.1186/s12913-019-4377-6 (PMC6734520; doi:10.1186/s12913-019-4377-6)
Supplement: Supplementary file 1 — Questionnaire on HDP and PPH practice in your facility. (PDF 108 kb) [file 12913_2019_4377_MOESM1_ESM.pdf]

Questionnaire on HDP and PPH practice in your hospital

Dear health care provider,

We kindly ask you to complete the following questionnaire for the purpose of a national assessment of the current management of hypertensive disorders in pregnancy and post partum hemorrhage. The completion of this questionnaire contributes to making the best possible national obstetric guidelines on these disorders. We would like your evaluation and feedback in all honesty.

Thank you for your contribution,

The maternal mortality committee

Age:	…
Sex:	male / female
Professional:	gynecologist / resident / intern / midwife / nurse / student
Years experience:	…
Hospital:	I / II / III / IV / primary health care / private sector
Department:	Delivery rooms / maternity ward / outpatient clinic / emergency department

General

1. Would you like national obstetric guidelines?
☐ yes ☐ maybe ☐ no

2. Would you like to help create the national obstetric guidelines?
☐ yes ☐ maybe ☐ no

3. Where would you like to find the national obstetric guidelines?
☐ delivery room ☐ guideline book ☐ website

4. Does your hospital have a local protocol on PPH or HDP?
☐ yes, both ☐ only HDP ☐ only PPH ☐ neither

Please answer the following questions according to the local reality in your institution.
Please use the space on this page to give additional comments and suggestions.

Post partum hemorrhage (PPH)

Definitions

1. Is there a clear definition for PPH?
 yes, > 1 liter
 yes, but the definition is as follows:
 no

2. Is there a clear definition for severe PPH?
 yes, > 1 liter
 yes, but the definition is as follows:
 no

3. Is it clear when you should alarm the doctor or your supervisor?
 yes, it is clear
 no clear rules

Prevention

4. Is oxytocin generally used in all caesarean sections?
 yes
 no

5. Is oxytocin generally used in all vaginal deliveries?
 yes
 only in the case of risk factors
 no

6. Is controlled cord traction performed?
 yes
 I do not know what that is
 no

Blood loss measurement

7. How is blood loss measured?
 in a measuring cup
 on a scale

8. What do you measure?
 everything, all the blood
 I throw away the first bit as it also contains amniotic fluid
 only the cloths

Medical treatment, in ongoing blood loss >500mL

9. Do you ever provide a second shot of oxytocin (i.v. or i.m,)
 yes
 only in infusion with crystalloid
 no

10. Do you treat her with oxytocin in crystalloids?
 yes
 no

11. Do you ever give additional misoprostol?
 yes
 no

12. Do you ever give additional Methergine?
 yes
 no

13. Do you ever give Tranexamic acid?
 yes
 no

Supportive treatment, in ongoing blood loss >500mL

14. Do you place a second i.v. line?
 yes
 only when necessary (for instance instable patien)
 no

15. Do you give crystalloids (sodium chloride / ringers lactate) for resuscitation?
 yes
 no

16. Do you give colloïds (Gelofusin) for resuscitation?
 yes
 no

17. Do you give oxygen?
 yes
 no

Blood transfusion in PPH

18. Are clear guidelines available for blood transfusion?
 yes
 no

19. When is blood transfusion generally given?
 Hb < 4 mmol/L
 Hb < 3.5 mmol/L and Ht <0.20
 Hb < 5 mmol/L
 Persistent blood loss

20. In what ratio are packed cells and fresh frozen plasma transfused?
 1 : 1
 2 : 1
 1 : 2
 Other, …

Other

21. Is a PPH box available with all necessary equipment?
 yes
 no

22. Have you ever used an intra-uterine balloon, B-lynch or uterine pack?
 yes
 no

23. Have you ever used a vaginal tampon?
 yes
 no

24. Does your facility perform a hysterectomy for severe PPH?
 yes
 no

25. Do you ever use the early warning score system (EWS / MEOWS)?
 yes
 no

Hypertensive disorders of pregnancy (HDP)

Definitions

1. Do you have a clear definition for pre-eclampsia?
- yes
- no

2. And for severe pre-eclampsia?
- yes
- no

3. And for eclampsia?
- yes
- no

Prevention

4. Do you and your colleagues know the most important risk factors for HDP?
- yes
- no

5. Does your facility give aspirin prevention?
- yes
- no

6. Does your facility give calcium prevention?
- yes
- no

Medical treatment

7. What is the 1st – 4th choice oral medication choice?
- methyldopa (Aldomet)
- hydralazine (Apresoline)
- nifidipine (Adalat)
- labetalol

Please note #1, #2, #3, #4 of most prescribed

8. What is the 1st – 4th choice i.v. medication choice?
- hydralazine in direct shots
- hydralazine in perfussor
- labetalol (Trandate)
- ketanserin

Please note #1, #2, #3, #4 of most prescribed

Magnesiumsulfate

9. Does your facility always give a loading dose?
- yes
- no

10. What is the loading dose?
- 1-2 grams in 30 minutes
- 4-6 grams in 30 minutes
- other:

11. What is the maintenance dose?
- 1 gram per hour
- ½ gram per hou
- other:

12. What is the duration of treatment? At least …
- 6 - 12 hours
- 24 - 48 hours
- other:

13. At what blood pressure threshold is $MgSO_4$ initiated?
- RR systolic:
- RR diastolic:

14. When a seizure occurs a few hours after initiation of $MgSO_4$, do you …
- double $MgSO_4$ dose to 2 grams per hour
- repeat bolus with 2 grams in 5 minutes
- other:

15. In eclampsia what medication is given first?
- magnesium sulfate
- diazepam
- antihypertensives
- other:

Stabilization of severe pre-eclampsia / eclampsia

16. When is pregnancy terminated in eclampsia?
- as soon as the patient is stable enough
- at least 48 hours after initiation of $MgSO_4$

17. From what gestational age is the pregnancy terminated by caesarean section if necessary?
- 27 weeks
- 30 weeks
- 32 weeks
- 34 weeks

Other

18. Is a HDP box available with all necessary equipment?
- yes
- no

19. Is oxygen given via non-rebreathing mask to a woman experiencing a seizure (eclampsia)?
- yes
- no

20. How many intravenous lines do you give a woman with severe pre-eclampsia or eclampsia?
- always one at least
- always two at least

21. Do you give patient fluids before initiation of magnesium sulfate?
- yes, at least 500mL
- yes, at least 1000mL
- no

22. Does a woman with pre-eclampsia or eclampsia have a fluid restriction?
- yes, …. mL per day
- no

23. Do you ever use the early warning score system (EWS / MEOWS)?
- yes
- no
