# Supplementary material for: Bottom-up development of national obstetric guidelines in middle-income country Suriname
Source: BMC Health Serv Res. 2019 Sep 9;19:651. doi: 10.1186/s12913-019-4377-6 (PMC6734520; doi:10.1186/s12913-019-4377-6)
Supplement: Supplementary file 2 — Comparison of hospitals in clinical practice of HDP and PPH in Suriname. (PDF 124 kb) [file 12913_2019_4377_MOESM2_ESM.pdf]

Hypertensive disorders in pregnancy – The variation in practice between the four hospitals in Paramaribo - Suriname

		Hospital A (2016)	Hospital B (2016)	Hospital C (2016)	Hospital D (2016)	The final national guideline
Definitions	**Pre-eclampsia**	*BPs ≥ 140 mm Hg **AND/OR** BPd ≥ 90 mm Hg (measured twice at least 4 hours apart) **OR** BPd ≥ 110 mm Hg; all after GA 20 weeks; **with** proteinuria*	BPd ≥ 90 (measured twice at least 4 hours apart) **OR** BPd≥ 110 **OR** increase of BP 15-20 compared to pre-pregnancy BP; all after GA 20 weeks; **with** proteinuria **OR** clinical symptoms	*BPs ≥ 140 **AND/OR** BPd ≥ 90 (twice; interval 4h) **OR** BPd ≥ 110; all after GA 20 weeks; **with** proteinuria*	BPs ≥ 140 **AND/OR** BPd ≥ 90 mm Hg (twice; interval 4h) **OR** BPd ≥ 110 mm Hg; all after GA 20 weeks; **with** proteinuria	BPs ≥ 140 mm Hg **AND/OR** BPd ≥ 90 (2x, interval 4 –6h) OR increase BPs >30 **OR** BPd >15 mmHg **with** proteinuria OR clinical symptoms
	**Severe pre-eclampsia**	*Pre-eclampsia with BP diastolic ≥ 110 mm Hg with clinical symptoms*	Not specified	*Pre-eclampsia with BP diastolic ≥ 110 mm Hg **OR** clinical symptoms*	Not specified	BPd ≥ 110 mmHg (once) **with** proteinuria **OR** organ dysfunction (renal, liver, hematological, neurological, pulmonal)
	**Eclampsia**	*Generalized tonic-clonic seizures, with no other cause than pre-existent pre-eclampsia*	Convulsion in a pre-eclamptic patient with no other cause	Convulsion in a pre-eclamptic patient with no other cause	Convulsion in a pre-eclamptic patient with no other cause	Tonic-clonic seizures with no other attributable cause than pre-eclampsia
	**Calcium**	*No*	History of IUGR or pre-eclampsia	No	No	Low intake → 2 grams in 2-3 trimester
	**Aspirine**	*Chronic hypertension or medical history of IUFD or IUGR due to pre-eclampsia*	History of IUGR or pre-eclampsia	No	No	High risk → 100mg in GA 16-37
	**Admission criteria**	BPs ≥ 160 **AND/OR** BPd ≥ 110 **AND/OR** clinical symptoms	Not specified	Not specified	Not specified	Severe PIH (persistent BPs ≥ 150/100), pre-eclampsie / HELLP / eclampsia, foetal indication
Hypertensive	**Oral initiation**	*BPd ≥ 100*	Not specified	BPd ≥ 100 mm Hg (twice)	BPd ≥ 100 **OR** BP increase >20	BPs ≥ 150 **OR** BPd ≥ 100
	**Parenteral initiation**	*BPd ≥ 110*	Not specified	BPd ≥ 110 mm Hg	BP diastolic ≥ 110 mmHg > once	BPd ≥ 110
	**Oral drug choice**<br>*Antepartum*<br><br>*Postpartum*	*1. methyldopa 250-750mg 2-3<br>2. hydralazine 25-50 2-3dd<br><br>nifedipine 20mg 2dd*	1. methyldopa 250-500mg 2-3<br>2. hydralazine 25-50 2-3dd<br><br>nifedipine 20mg 2dd	1. methyldopa 250-500mg 2-3<br>2. hydralazine 25-50 2-3dd<br>3. labetalol 100-200mg 2dd<br>nifedipine 20mg 2dd	1. methyldopa 250-500mg 2-3<br>2. hydralazine 25-50 2-3dd<br><br>nifedipine 20mg 2dd	1. methyldopa 250-1000mg 3-4dd<br>2. hydralazine 25-75mg 3dd<br>3. labetalol 50-200mg 3dd<br>nifedipine 5-40mg 3dd
	Parenteral drug choice	*1. hydralazine via perfusor<br>2. hydralazine 5mg i.v.<br>3. ketanserine or labetalol<br>Perfusor available*	1. hydralazine via perfusor<br>2. hydralazine 5mg i.v.<br>3. labetalol<br>Perfusor available only in ICU	1. hydralazine via perfusor<br>2. hydralazine 5mg i.v.<br>Perfusor available	hydralazine 5mg i.v.<br>Perfusor not available	1. hydralazine 5-10mg à 20-40 min. **OR** by infusion system<br>2. labetalol 1-2 mg/kg in 20 min. Continue 20-70mg/hr.
$MgSO_4$	**Indication**	*Eclampsia; severe pre-eclampsia; neuroprotection*	Eclampsia; pre-eclampsia	Eclampsia; severe pre-eclampsia	Eclampsia; severe pre-eclampsia; pre-eclampsia with symptoms	Eclampsia; severe pre-eclampsia (BPd ≥ 110 OR clinical symptoms); neuroprotection
	**Loading dose**	*4 grams / 30 minutes*	2 grams / 30 minutes	4-6 grams / 30 minutes	1 gram / 30 minutes	4-6 grams / 30 minutes
	**Maintanance dose**	*1 gram / hour*	1 gram / hour	1 gram / hour	1 gram / hour	1 gram / hour
	**Dose repeated seizure**	*2 grams / 5 minutes*	None (diazepam)	None (diazepam)	None (diazepam)	2 grams / 5 minutes
	**Stop treatment**	After 48 hours	After 24 hours and stable	After 48 hours	After 48 hours and stable	24 – 48 hours (max 7 days; measure plasma-$MgSO_4$)
	**Diazepam indication**	If no iv-access	If no iv-access or in repeated seizure	If no iv-access or in repeated seizure	In seizures (whether or not on $MgSO_4$)	If no iv-access; when $MgSO_4$ shot given twice
	**Oxygen**	*Eclampsia: 10 L NRM*	No	Eclampsia: 5-10 L NRM	No	Eclampsia: 15 L NRM
	**Intravenous access**	*1 in pre-eclampsia; 2 in eclampsia*	1 in pre-eclampsia and eclampsia	1 in pre-eclampsia; 2 in eclampsia	1 in pre-eclampsia and eclampsia	2
Fluids	**Before i.v. drugs**	500mL NaCl 0.9% quick	1000mL gelofusine quick	500mL Ringers **OR** gelofusine	No fluids	No fluids
	**Maintanance**	500mL Ringers à 8 hours (no gelofusine, glucose/NaCl 0.9%, glucose 5%)	500mL glucose/NaCl 0.9% à 6 hours	First 1000mL Ringers in 6 hours, then 500mL gluc/NaCl 0.9% à 8 hours	500mL Ringers **OR** NaCl 0.9% à 8 hours	Max. 1500mL in 24 hours.<br>Preference for Ringer's Lactate or NaCl 0.9%
Monitoring	**Vital signs**					
	RR <110	Every 6 hours	Every 4 hours	Every 2-3 hours	Every 6 hours	Every 4 hours (intrapartum every ½ hour)
	RR ≥ 110 OR $MgSO_4$	4x once per hour, then every 4 hours	Every 2-3 hours	4x every ½ hour, 4x every hour	Every 4 hours	Every ½ hr (4x), 1hr (4x), 2 hrs (4x), 4 hrs (4x)
	**Fluid balance**	*Pre-eclampsia*	Pre-eclampsia / HELLP	Severe pre-eclampsia	Pre-eclampsia	Every admission of hypertensive disorders
	**Blood tap**	*2x per week*	2x per week	2x per week	3-4x per week	2x per week
	**Doppler**	*GA <27*	GA <34	GA <30	GA <30	GA <27
	**CTG**	*GA ≥27*	GA ≥34	GA ≥30	GA ≥30	GA ≥27
	**Corticosteroids**	*GA 26^{+5} – 34<br>Dexamethasone 12mg twice; 12hrs*	Severe pre-eclampsia and GA 28-34<br>Dexamethasone 12mg twice; 12hrs	Delivery expected and GA 27-34<br>Dexamethasone 12mg twice; 12hrs	Severe pre-eclampsia and GA 28-34<br>Dexamethasone 12mg twice; 12hrs	GA 26 – 34<br>Dexamethasone 4 x 6mg; 12 hours apart (total 36 hours)
	**Eclampsia box**	*In two delivery rooms*	Not present	In one delivery room	Not present	In every delivery room
Delivery	**Timing mild PE**	GA 37<br>Induction (balloon, misoprostol)	Dependent on gynaecologist<br>Induction (misoprostol) or C-section	GA 37, induction<br>Induction (balloon, misoprostol)	GA 37<br>Induction (misoprostol) or C-section	GA 37; preference for vaginal birth with induction (depending on BISHOP → balloon/misoprostol/oxytocine)
	**Timing severe PE**	GA ≥27<br>C-section atleast >48 hours of stabilisation	GA ≥34<br>C-section after stabilisation	GA ≥30<br>C-section / induction after stabilisation	GA ≥32<br>C-section after stabilisation	GA <27 induction. GA ≥ 27: preference for induction. Caesarian in case of failed induction or fetal indication
Postpartum	**Duration of admission**	At least 24 hours post partum	At least 24 hours post partum	At least 24 hours post partum	At least 24 hours post partum	At least 24 hours post partum and stable (BP normalized, urine output normal, lab results improved)
	**Antihypertensives**	No target BP; switch to nifidepine	No target BP; switch to nifidepine	No target BP; switch to nifidepine	No target BP; switch to nifidepine	BPs 130-150 and BPd 80-100; switch to nifidepine
	**Blood tap**	If necessary	One day post partum	If necessary	Two days post partum	One day post partum
	**Postnatal clinic visit**	7 days	7-10 days	7 days	7 days	7-10 days and 6 weeks

Postpartum hemorrhage – The variation in practice between the four hospitals in Paramaribo - Suriname

		Hospital A (2016)	Hospital B (2016)	Hospital C (2016)	Hospital D (2016)	The final national guideline
	**Definition PPH**	500mL blood loss or more<br>after vaginal delivery	500mL blood loss or more<br>after vaginal delivery	500mL blood loss or more<br>after vaginal delivery	500mL blood loss or more<br>after vaginal delivery	500mL blood loss or more during and after childbirth
	**Definition severe PPH**	1000mL blood loss or more	Not specified	Not specified	Not specified	1000mL blood loss or more ór blood loss with clinical hypovolemia during and after child birth
	**Call for help (supervisor)**	500mL blood loss or more (general doctor)<br>1000mL blood loss or more (gynaecologist)	Not specified	500mL blood loss or more	500mL blood loss or more	500mL blood loss or more (call general doctor)<br>1000mL blood loss or more (call gynaecologist)
	**Risk factors**	Not specified	Prolonged labor, twin pregnancy, macrosomia, high parity, uterus myomatosis	Not specified	Not specified	Risk factors grouped (see guideline for elaboration):<br>- General (e.g. age >40, BMI >35, uterus myomatosis)<br>- Obstetric history (e.g. grande mult, caesarean, etc)<br>- Current pregnancy (macrosomia, pre-eclampsia, etc)<br>- During delivery (prolonged labor, etc)<br>- Post partum (full bladder, retained placenta, etc).
**Prevention**	**Partograph**	On indication (prolonged labor, caesarean in history, on request of physician)	Sometimes	Never	Never	Always recommended
	**Oxytocin in the 3rd stage of labor**	Caesarean – always<br>Vaginal birth – always (before delivery of placenta)<br>5-10 IU i.m. or slowly i.v. (2 minutes)	Caesarean – always<br>Vaginal birth – in patients with high-risk for PPH (done after delivery of placenta)<br>5-10 IU i.m. or slowly i.v. (2 minutes)	Caesarean – always<br>Vaginal birth – in patients with high-risk for PPH (done after delivery of placenta)<br>5-10 IU i.m. or slowly i.v. (2 minutes)	Caesarean – always<br>Vaginal birth – in patients with high-risk for PPH (done after delivery of placenta)<br>5-10 IU i.m. or slowly i.v. (2 minutes)	Caesarean – always<br>Vaginal birth – always (before delivery of placenta)<br><br>5-10 IU i.m. or slowly i.v. (2 minutes)
	**Measurement of blood**	- Measurement cup (mL)<br>- Liquid (blood) removed from blood clots with a sve. Measurement done of only the blood clots.	- Measurement cup (mL)<br>- Liquid (blood) removed from blood clots with a sieve. Measurement done of only the blood clots.	- Measurement cup (mL) and / or scale (kg)<br>- Liquid (blood) and blood clots measured	- Measurement cup (mL)<br>- Liquid (blood) removed from blood clots with a sieve. Measurement done of only the blood clots.	- Measurement cup (mL) and / ore scale (kg)<br>- Measure liquid (blood) and bloodclots all together. Subtract the estimated amniotic fluid.
**Management**	**Oxytocin i.m. / i.v.**	Repeat 5-10 IU shot i.m. or i.v.	5 IU shot i.m. or i.v.	5 IU shot i.m. or i.v.	10 IU shot i.m. or 5 IU shot i.v.	10 IU shot i.m. or i.v.
	**Oxytocin infusion**	10 IU in 500mL / 4-6 hours<br>During at least 24 hours	5 IU in 500mL / 6 hours<br>Until bleeding stops	10-20 IU in 500mL / 4 hours<br>During at least 4 hours	10 IU in 500mL / 4-6 hours<br>Until bleeding stops	10 IU in 500mL / 4 hours
	**Misoprostol**	400 – 800 micrograms rectal	400 – 800 micrograms rectal	400 micrograms rectal	400 – 800 micrograms rectal	Not first choice, as misoprostol is not beneficiary when adequate oxyotin dosis is given.
	**Methergin**	Not prescribed	0.2 mg i.m. if bleeding persists	Not prescribed	Not prescribed	0.2 mg i.m.
	**Tranexamic acid (Cyclokapron)**	1 gr i.v. slowely in persistent bleeding	2 gr i.v. slowely in severe and persistent bleeding	2 gr i.v. slowely in severe and persistent bleeding	Not prescribed	1 gram i.v. slowely when 1000mL blood loss or more
	**Calcium gluconate**	1 gram i.v.<br>After transfusion of 4 blood products	1 gram i.v.<br>After transfusion of 6 blood products	1 gram i.v.<br>After transfusion of 4 blood products	Not prescribed	1 gram i.v.<br>After transfusion of 4 blood products
**Rescuscitation**	**I.V. access**	2 i.v. access lines (> 1000 mL blood loss)	Not specified	Not specified	Not specified	2 x intravenous access (preferably green or grey)
	**Infusion**	Ringer's Lactate or Sodium Cloride 0.9%	Ringer's Lactate or Glucose 5%	Sodium Cloride 0.9%, Glucose 5% or Gelofusine	Ringer's Lactate or Sodium Cloride 0.9%	Ringer's Lactate or Sodium Cloride 0.9%<br>Infuse 1 : 1 (blood loss : infusion), max 3 liters
	**Oxygen**	10 L / min in 1000 mL blood loss or more	Sometimes in severe blood loss	10 L / min in 2000 mL blood loss or more	Sometimes in severe blood loss	10 – 15 L / min in 1000 mL blood loss or more
	**Transfusion indication**	Hb < 4 mmol/L	Hb < 5 mmol/L	Hb < 3.5 mmol/L or Ht < 0.20	Hb < 4.5 mmol/L and complaints	Hb < 4.0 mmol/L or Ht < 0.20, 1000 mL blood loss or more and persistent or with symptoms of hypovolemic shock
	**Blood products ratio**	2 PC : 1 FF	2 PC : 1 FFP	1 PC : 2 FFP	2 PC : 1 FFP	1 PC : 2 FFP (in massa transfusion 4 PC : 4 FFP : 1 TC)
	**Surgical and other**	Vaginal tampon; Hysterectomy	Vaginal tampon; Hysterectomy	Vaginal tampon; Hysterectomy	Vaginal tampon; Hysterectomy	Vaginal and uterine packing (gauzes); balloon tamponade (Bakri, Foley, condom); B-lynch; bilateral ligation of uterine (or internal iliac) arteries; hysterectomy (rather sooner than later)
**Vital signs**	**Measurements of**	Blood pressure, pulse, temperature , blood loss, tone of uterus	Blood pressure, pulse, temperature, blood loss, tone of uterus	Blood pressure, pulse, temperature, blood loss, tone of uterus	Blood pressure, pulse, temperature, blood loss, tone of uterus	Blood pressure, pulse, **respiratory rate, saturation,** temperature, blood loss, tone of uterus, **urinary output**
	**Timing of monitoring**<br>- after a normal delivery<br>- during hemorrhage<br>- after hemorrhage	 Directly; after 1 hour; after 8 hours<br>Not specified<br>Directly; after 1 hour; after 4 hours	 Directly; after 1 hour; after 8 hours<br>Not specified<br>Directly; after 1 hour; after 8 hours	 Directly; after 1 hour; after 8 hours<br>Not specified<br>Directly; after 1 hour; after 2 hours	 Directly; after 1 hour; after 8 hours<br>Not specified<br>Directly; after 1 hour; after 8 hours	 Directly; after 1 hour; after 8 hours<br>Every 10 minutes<br>Directly; after 30 minutes; 1 hour; 2 hours; 8 hours
	MEOWS score performed	Yes	No	No	No	Not specified
	**Follow up in severe PPH**	- Blood exam (Hb, Ht, Tr, INR, fibrinogen)<br>- Performed a day later<br>- Discharge not specified	- Blood exam (not specified)<br>- Performed same day<br>- Discharge not specified	- Blood exam (not specified)<br>- Performed a day later<br>- Discharge not specified	- Blood exam (not specified)<br>- Performed same day<br>- Discharge not specified	- Blood exam (Hb, Ht, Tr, INR, APTT, PT fibrinogen, electrolytes, creatinine and liver enzymes)<br>- Performed 4 hours later and 24 hours later<br>- Discharge (by doctor) after at least 24 hours
	**PPH-box in delivery room**	Yes	No	Yes	No	Yes, should always be
	**PPH-checklists or flow charts in delivery rooms**	Yes	No	Yes	No	Yes, should always be
