# Supplementary material for: Bottom-up development of national obstetric guidelines in middle-income country Suriname
Source: BMC Health Serv Res. 2019 Sep 9;19:651. doi: 10.1186/s12913-019-4377-6 (PMC6734520; doi:10.1186/s12913-019-4377-6)
Supplement: Supplementary file 3 — Comparison of international guidelines regarding HDP and PPH. (PDF 124 kb) [file 12913_2019_4377_MOESM3_ESM.pdf]

Hypertensive disorders of pregnancy
Summary of definitions, prevention and management of four international guidelines

	WHO guideline (2011)	ACOG-guideline (2013)	Queensland Brisbane (2016)	NVOG-guideline (2011)
**Definition**				
**- Pre-eclampsia**	Onset of new episode of hypertension during pregnancy (persistent diastolic BP ≥ 90 mm Hg) with substandial proteinuria (>0.3 g/day)	BP systolic ≥ 140 and/or diastolic ≥ 90 mm Hg (measured twice at least 4 hours apart) after GA20 **OR** BP systolic ≥ 160 and/or diastolic ≥ 110 mm with either proteinuria **OR** with severity symptoms	Hypertension (BPs≥ 140 OR BPd ≥ 90) after 20 weeks of gestation on 2 or more occassions accompanied by one of the following: proteinuria (>30mg/mmol), creat >90, oliguria, thrombopenia (<100), hemolysis, raised transaminases, DIC, neurological symptoms, pulmonary oedema or fetal growth restriction.	Pregnancy induced hypertension (BP systolic ≥ 140 **and/or** diastolic ≥ 90 mm Hg after GA 20, meassured twice in women with normal previous BP. BP should be normal within 3 months post partum)<br><br>with proteïnuria (>0.3g/day)
**- Severe PE**	Severe hypertension (not defined), heavy proteinuria or substantial maternal organ dysfunction. Early onset (before 32-34 weeks) and fetal morbidity are used as independent severity criteria in some countries	Systolic ≥ 160 or diastolic ≥ 110 mm Hg (measured twice at least 4 hours apart while patient is on bed) **AND** thrombopenia <100 **OR** impaired liverfunction or epigastric pain **OR** impaired renal function (creatinine 2x normal) **OR** pulmonary oedema **OR** cerebral/ visual disturbances.	[Magpie Trial] BPs ≥ 170 or BPd≥ 110 mm Hg **AND** 3+ proteinuria **OR** BPs ≥ 150 or BPd≥ 100 mm Hg **AND** 2+ proteinuria **AND** 2 severity symptoms **OR** pre-eclampsia with at least one sign of central nervous system irritability	Systolic ≥ 160 or diastolic ≥ 110 mm Hg **OR** clinical symptoms of pre-eclampsia (headache, upper-abdominal pain, nausea) **OR** proteïnuria >5 g / 24 hours. [Proteinuria can remain absent]
**- Eclampsia**	Generalized seizures in addition to pre-eclampsia criteria	New-onset grand mal seizures in a woman with pre-eclampia	One or more seizures superimposed on PE (HT/proteinuria may be absent)	Not specified
**Prevention**				
**- Calcium**	When low calcium intake (<900mg/ day)	In populations with low calcium intake	In high risk women with low calcium intake	Not specified
**- Aspirin**	75mg/ day in high risk women, initiate GA<20 weeks	In high risk women, start 60-80 mg daily in late first trimester	Moderate to high risk of PE; 100mg/day , initiate <16 weeks. Until 37 weeks or birth of baby (NNT 42)	Not specified

	WHO guideline (2011)	ACOG-guideline (2013)	Queensland Brisbane (2016)	NVOG-guideline (2011)
**Medical treatment**				
**Antihypertensive**				
**-- Initiation**	Not specified	Persistent BP systolic ≥ 160 mm Hg **OR** diastolic ≥ 110 mm Hg	BP systolic ≥ 160 **OR** diastolic ≥ 100 mm Hg	Systolic ≥ 160 **OR** diastolic ≥ 110 mm Hg
**-- Drug choice**	Hydralazine, methyldopa, labetalol, nifedipine, ketanserin, and others are compared. No preference or clear recommendation.	Labetalol, nifedipine, methyldopa	Oral: methyldopa, labetalol, oxyprenolol, hydralazine, nifedipine, prazosin, clonidine.<br>I.V. nifedipine, hydralazine, labetalol, diazoxide	Methyldopa, labetalol, nifedipine
**Magnesium sulfate**				
**-- Indication**	Eclampsia; severe PE	Eclampsia; severe PE	Eclampsia; HELLP; severe PE; neuroprotection	Eclampsia; severe PE, consider in mild pre-eclampsia
**-- Loading dose**	Not specified	4-6 (time ?)	4 g IV in 20 min	4-6 g in 10-30 min
**-- Maintanance**	Not specified	1-2 g per hour	1 g per hour	1 g in 60 min
**-- 2nd seizure**	Not specified	No specific recommendations	2 g in 5 min (may repeat after 2 min) Other possibilities: diazepam 5-10mg IV (2-5mg/min), midazolam 5-10 mg IV in 2-5min	2 g in 5 min (max. twice) Other possibilities: lorazepam 4mg i.v. slowly, sedate and intubate
**-- Stop treatment**	Not specified	At least 24 hours after last convulsion	At least 24 hours after birth or last seizure	At least 24 hours after initiation
**Other**				
**Corticosteroids**	Not specified	GA <34 weeks	GA <34 weeks	GA <34, birth expected in 2-10 days; Betamethason 12mg i.m. 2x (24hr apart)
**Vital signs**	Not specified	Every 8 hours	At least every 4 hours	Not specified
**Fluids**	Not specified	Not specified	No large volumes of fluids. Restrict to 1.5L/24 hrs after birth. Strict fluid balance	Not specified
**Delivery timing in severe PE**	Unviable: induction.<br>Term: delivery asap<br>Before term: expectant unless uncontrolled hypertension or fetal distress	Depends on severity; GA and NICU-facility.<br>Deliver shoud be shortly after stabilisation.	Stabilize (control hypertension, correct coagulopathy, initiate $MgSO_4$, control fluids) and deliver	Not specified
**Post partum medication**	Not specified. Severe post partum hypertension; in all women treated antenatally	Not specified. Initiate when BP systolic ≥ 150 OR diastolic ≥ 100 mm Hg.	Avoid abrupt withdrawal of antihypertensives. Cease methyldopa (depression). Consider nifedipine.	Not specified
**Other recommendations**	Not specified	No NSAIDS, promote breastfeeding. Consider postnatal counseling.	Consider VTE profylaxis (during admission); postnatal counseling. Follow-up 6 weeks	Not specified

Legend: BP = blood pressure, GA = gestational age, HT = hypertension, IV = intravenous, MgSO4 = magnesium sulfate, NNT = number needed to treat, VTE = venous thrombo-embolism, NSAID = non-steroidal anti-inflammatory drugs.

Postpartum hemorrhage
Summary of definitions, risk factors, prevention and resuscitation of four international guidelines

	WHO-guideline (2011)	ACOG-guideline (2013)	RCOG-guideline (2014)	NVOG-guideline (2011)
**Definition**	PPH: >500mL<br>Severe PPH >1 L	Vaginal > 500mL<br>Caesarean >1 L	Minor: >500mL<br>Moderate: 1-2 L<br>Major: >2 L	Vaginal > 500mL<br>Caesarean >1 L
**Incidence**	2%	4-6%	3.7/1000 (> 5 PC)	3.8% (2003)<br>6.2% (2009)
**Risk factors**	Minimally described	Elaborately described	Elaborately described with odds ratio	Elaborately described
**Prevention**	AMTSL<br>(CCT after 30 min)	Not discussed	AMTSL<br>Placenta location	AMTSL
- Oxytocin	10 IU i.m. / i.v. in all births	Not specified	Oxytocin 5 or 10 IU i.m.<br>5 IE i.v. (Caesarean)<br>Ergometrin 0.5 mg i.m.<br>Combined if Hb low	Oxytocin 5 or 10 IU i.m.<br>5 IE i.v. (Caesarean)<br>followed by 10 IU i.v. in 4 hours
**Resuscitation**				
- Access	Not specified	Ample intravenous access	Intravenous access 2x	Intravenous access 2x
- Fluids	Crystalloids	Crystalloids	Crystalloids, warm/ rapid<br>Max. 2 L (+ 1.5L colloids)	Crystalloids, warm/ rapid (1 : 1 blood loss)
- Oxygen	Not specified	10-15 L per minute	10-15 L / min over NRM	Not specified
- Blood products	Not specified	Blood as needed and blood bank notification	As soon as possible<br>6 PC : 4 FFP	4 PC : 4 FFP
- Uterine massage	Recommended, contin-uously. Bimanual, aorta compression advised	Not specified	Not specified	Recommended, continuously
- Coagulation screening	Blood loss up to 1500mL or >2 uterotonics	Not specified	>1L blood loss	>1L blood loss or >2L cristalloids
**Medical treatment**				
- Oxytocin infusion	Recommended	10-40 IU i.v. or 10 IU i.m.	5 IU i.v. may repeat or 40 IU in 500mL à 4 hrs	10 IU i.v. followed by 10 IU i.v. à 4 hrs
- Misoprostol	If oxytocin not available: 600ug oral or 800ug sublingual	800-1000ug rectal	1000 ug rectal	Not recommended
- Ergots	If oxytocin not available<br>Not in retained placenta	Methyl-ergonovine 0.2 mg i.m. every 2-4 hr	Ergometrine 0.5mg i.m. or i.v.	Metergin 0.2mg i.m or i.v.
- Others	Not specified	Carboprost 0.25mg i.m.<br>Dinoprostone 20mg PV<br>Factor VIIa 50-100ug/kg every 2 hours	Carboprost 0.25mg i.m. (max 8 doses)	Sulproston 500 ug / 30 mins (followd by 60-120 ug per hour). Fibrinogen 2g (in >2L blood loss)
- Tranexamic Acid	Recommended in case of persistent bleeding	Not specified	Not recommended	Recommended, 1-2 g
- Antibiotic profylaxis in MPR	Ampicillin or cefazolin	Not specified	Not specified	Cefazolin/metronidazol or amoxicillin/clav. acid
**Surgical treatment**				
- Uterine packing	Not recommended	4-inch gauze 5000U thrombin in 5mL saline	Not recommended	Not recommended
- Balloon tamponade	Refractory bleeding/ Uterotonics unavailable	Foley (60-80mL)<br>Bakri (300-500mL)	Leave for 4-6 hrs	In refractory bleeding<br>Bakri (300-500mL)
- Brace suture	Not specified	B-Lynch, square	B-Lynch, square	B-Lynch, square
- Vessel ligation	Uterine and iliac artery	Uterine and iliac artery	Uterine and iliac artery	Not specified
- Embolization	If bleeding stable	Not specified	Yes, consider	Yes, consider
- Hysterectomy	In refractory bleeding despite vessel ligation	Not specified	Rather sooner than later	In case of placenta accreta, uterine rupture or Jehova's witnesses

Legend: AMTSL = active management of the third stage of labor. CCT = controlled cord traction. FFP = fresh frozen plasma. Hb = hemoglobin. MPR = manual placenta removal. NRM = non-rebreathing mask. PC = packed cells.
